# The Promise of Genomic Studies on Human Diseases: From Basic Science to Clinical Application

**DOI:** 10.1155/2017/5093167

**Published:** 2017-03-29

**Authors:** Lam C. Tsoi, Bethany Wolf, Y. Ann Chen

**Affiliations:** ^1^Department of Dermatology, University of Michigan Medical School, Ann Arbor, MI, USA; ^2^Department of Computational Medicine & Bioinformatics, University of Michigan Medical School, Ann Arbor, MI, USA; ^3^Department of Biostatistics, University of Michigan, Ann Arbor, MI, USA; ^4^Department of Public Health Sciences, Medical University of South Carolina, Charleston, SC, USA; ^5^Biostatistics and Bioinformatics, Moffitt Cancer Center, Tampa, FL, USA

## 1. Introduction

The advances in biotechnologies and efficiency in computational resources have provided unprecedented opportunities to study and analyze the genomics of human diseases. Over the last decade, high-throughput experiments studying -omics (e.g., genetics, epigenetics, or transcriptomics) have been used to generate informative data researchers can use to test different data-driven hypotheses. A big promise of such high-dimensional -omics data is the advancement of biomedicine by effectively translating findings from basic science research into clinical application. Designing and conducting genomic experiments in biomedical research aim to enhance the diagnosis, treatment, and prevention of human diseases. Translating -omics findings into clinical practice requires a flexible framework to incorporate different -omics data types to predict clinical outcomes in an integrated fashion.

## 2. Data Analysis

Developing rigorous statistical approaches and implementing innovative computational tools play essential roles in translating the findings based on high-dimensional -omics data into accurate and informative medical decisions. To equip readers with updated analytical approaches, this special issue covers a wide range of analytical approaches and pipelines. J. D. Hintzsche et al. provided a comprehensive review of computational tools to analyze and interpret the whole exome sequencing (WES) data, including alignment, variant calling, and annotation approaches developed for “pre-VCF (variant calling file)” analyzes, as well as major approaches to conduct downstream analysis after VCF file has been generated: pathway analysis, somatic prediction, copy number estimation, and so forth. Robustness and the ability to replicate findings in independent datasets are also critical in analyzing high-dimensional data. For analyzing transcriptomic data, A. E. Berglund et al. proposed a principal component analysis- (PCA-) based technique to reveal gene expression signatures that are robust in replicated datasets. The method can also identify complex signatures from independent biological components. Beyond traditional data analysis, W. Wei et al. demonstrated that visualization is a key component to translate -omics data into useful information. The study utilized the GPA (genetic analysis incorporating pleiotropy and annotation) and MDS (multidimensional scaling) techniques to illustrate genetic relationships between different human traits/diseases, revealing the underlying shared genetic architecture.

## 3. Data Integration

Data integration is essential for robust modeling of complex or heterogeneous conditions. By integrating gene expression data with prior biological knowledge such as tissue of origin or mutation status, V. P. Kamath et al. illustrated enhanced performance on radiation sensitivity. Their results provide a proof of concept on how accounting for biological heterogeneity can lead to robust modeling of clinical response. D. M. Rotroff and A. A. Motsinger-Reif reviewed current data integration techniques for joint-analysis of multiple -omics data and discussed future directions and challenges for applying these integrative approaches in personalized medicine. K. Raja et al. then discussed how researchers can utilize the large volume of data from the literature to develop biological inference for -omics analysis by providing an in-depth review of text-mining approaches that can be used to synthesize biomedical or clinical information and also highlighted the applications of text-mining in genomic, proteomic, and transcriptomic studies.

## 4. Biological and Clinical Inference

A. Muthiah et al. proposed a novel inference technique, called Module Anchored Network Inference (MANI), to reveal gene-gene relationships and provide inference on disease mechanism by using time-series gene expression data on adipocyte differentiation. Instead of utilizing all candidate genes from data, the MANI approach constructs small network modules, which is shown to outperform other in silico network inference techniques. Many human diseases are heterogeneous in nature and thus are challenging to provide accurate diagnosis and monitoring. Using systemic lupus erythematosus as a disease model, E. Zollars et al. applied a genomic technique to develop robust biomarker signature to better monitor disease activity. The approach utilized gene expression profiles to guide the classification of patients with different disease activities.

## 5. Conclusion

This special issue presents and discusses technological and methodological developments in biomedical research leading to advances in biomedicine through analysis and evaluation of -omics data. The research and review articles provide a comprehensive collection of approaches and studies for translating biological information from high-dimensional data to clinical applications. With the explosion of big data, we believe that innovative techniques, rigorous analytical approaches, and pipelines are keys to provide robust findings that can advance their clinical applications.

